# Gangrène ischémique du membre supérieur corrélée à l’infection de la COVID-19: à propos de 2 cas

**DOI:** 10.11604/pamj.2023.44.77.37507

**Published:** 2023-02-07

**Authors:** Papa Amadou Ba, Abdoulaye Ba

**Affiliations:** 1 Service d'Orthopédie-Traumatologie, Hôpital d'Instruction des Armées Principal, Dakar, Sénégal

**Keywords:** Ischémie, gangrène, COVID-19, cas clinique, Ischemia, gangrene, COVID-19, case report

## Abstract

Les manifestations cliniques de la COVID-19 ont beaucoup évolué allant de signes respiratoires et Oto-rhino-laryngologie (ORL) aux complications extra pulmonaires thrombotiques, neurologiques, cardiaques, rénales. Nous rapportons les observations de deux patients atteints de pneumopathie SARS-CoV-2 et dont l'évolution a été marquée par une ischémie dépassée du membre supérieur. L'association de complications thrombotiques veineuses, mais aussi artérielles, à l'infection virale est maintenant bien établie, et semble en rapport avec une hypercoagulabilité.

## Abstract (en)

Clinical manifestations of COVID-19 have changed a lot, ranging from respiratory and Ear, Nose and Throat (ENT) symptoms to extra pulmonary thrombotic, neurological, cardiac and renal complications. We here report the case of two patients with SARS-CoV-2 pneumonia whose course was marked by prolonged upper limb ischemia. The association between venous, but also arterial, thrombotic complications and viral infection is now well established, and appears to be related to hypercoagulability.

## Introduction

Apparue en 2019 en Chine la COVID-19 a touché tous les pays du monde. Au Sénégal, le premier cas était diagnostiqué le 02 mars 2020 et le premier décès était survenu le 02 avril 2020. Depuis lors il s'en est suivi plusieurs cas avec plusieurs décès. Les manifestations cliniques ont beaucoup évolué allant de signes respiratoires et ORL aux complications extra pulmonaires thrombotiques, neurologiques, cardiaques, rénales [1]. Aujourd'hui il est avéré qu'elle touche tous les organes avec des complications variées. Les manifestations cardio-vasculaires sont nombreuses et variées. Entre autre l'hypercoagulabilité et l'état de pro-inflammation concourent à des atteintes cardiovasculaires telles que les troubles thromboemboliques qui sont péjoratives [2]. Nous rapportons deux cas d'ischémie de membre supérieur chez des patients atteints de COVID-19 qui sont arrivés tardivement dans un centre hospitalier.

## Patient et observation

### Observation 1

**Figure 1:**
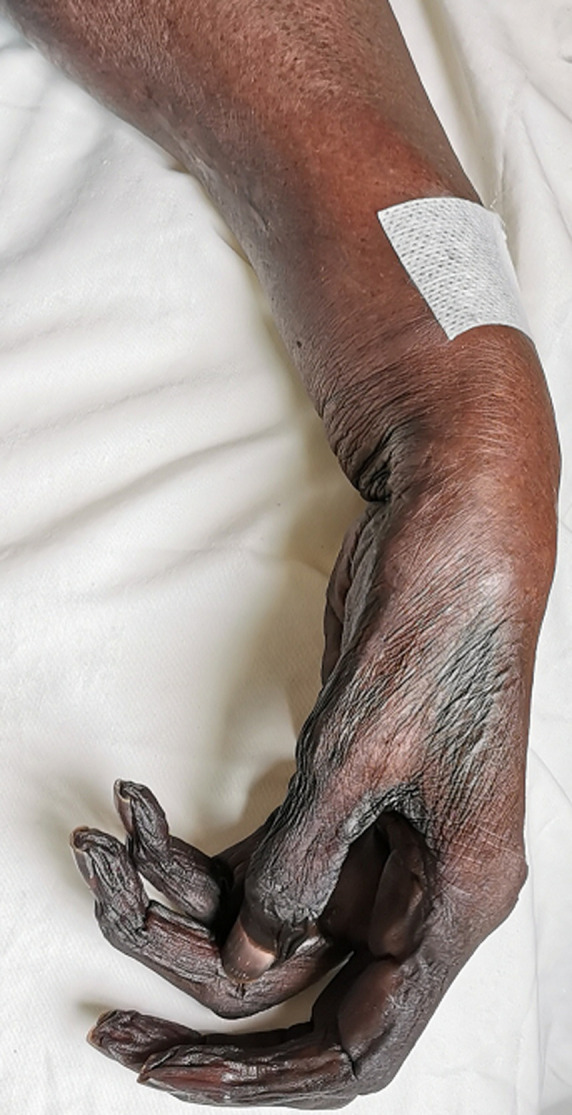
gangrène ischémique de la main du patient 1

**Présentation du patient:** il s'agissait d'une femme de 76 ans sans antécédents pathologiques rapportés qui a été admise aux urgences pour une ischémie dépassée du membre supérieur gauche (Figure 1).

**Résultats cliniques:** elle présentait à l'arrivée des signes de détresse respiratoire avec une SpO2 à 92 à l'air ambiant, une polypnée à 26 cycles par minute.

**Chronologie:** le début remonterait à environ 1 semaine par un syndrome grippal et douleur du membre supérieur qui l'a amené à consulter dans un centre de santé.

**Figure 2:**
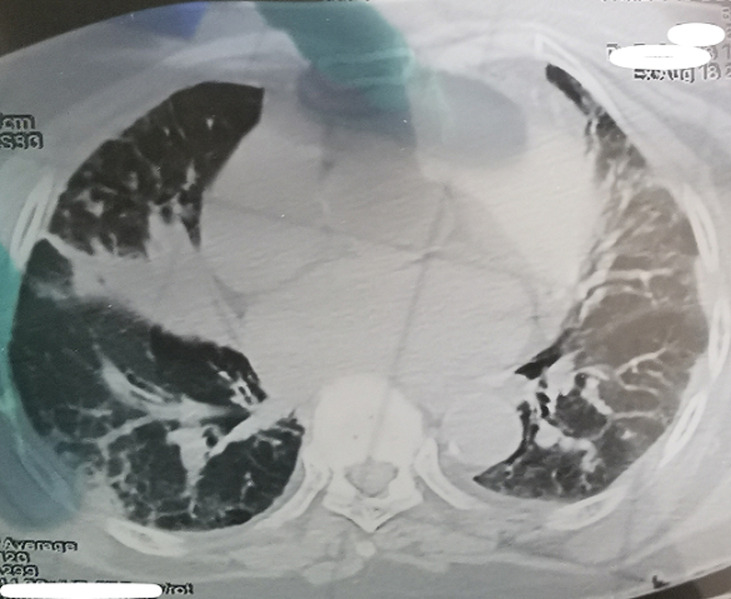
TDM pulmonaire montrant des lésions parenchymateuses

**Figure 3:**
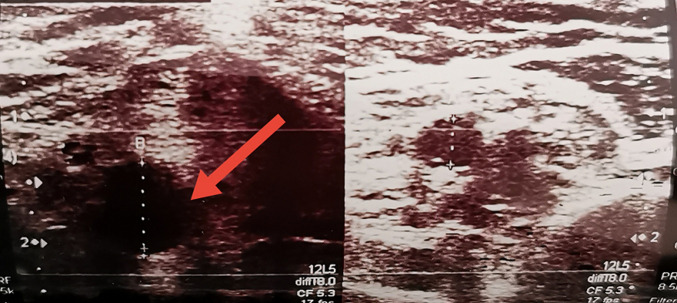
échodoppler; artère non compressible

**Démarche diagnostique:** la tomodensitométrie thoracique réalisait en urgences était en faveur d'une pneumopathie liée au SARS-CoV 2 avec atteinte de 25% du parenchyme pulmonaire (Figure 2). L'échodoppler artériel du membre supérieur fait en urgence avait montré une thrombose extensive de l'artère brachiale gauche (Figure 3). Devant ce tableau il a été admis à l'unité COVID. Le test *Polymerase Chain Reaction (PCR)* réalisé était positif.

**Traitement, suivi et résultats:** il a été mis sous traitement à base d'hydroxychloroquine, azythromycine, méthylprednisone et une supplémentation en zinc. Le délai pour la revascularisation était dépassé pour la gangrène. Une amputation de bras a été réalisée. Le traitement a été complété par une antibiothérapie efficace. Il est ensuite admis en unité de traitement COVID-19 pour le reste de la prise en charge.

**Consentement du patient:** les auteurs certifient avoir obtenu un consentement oral et signé du patient pour que les images et autres informations cliniques soient rapportées dans le journal. Nous nous abstiendrons à dissimuler son identité mais l'anonymat ne peut être garanti.

### Observation 2

**Figure 4:**
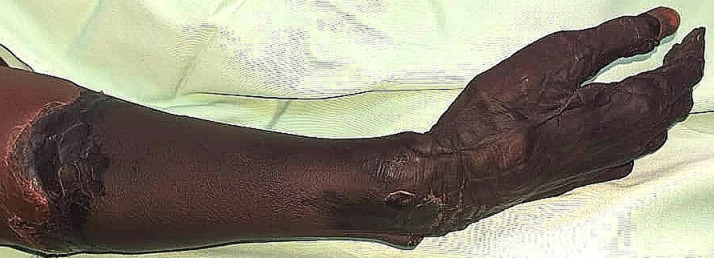
ischémie de la main et de l´avant-bras du patient 2

**Présentation du patient:** il s'agissait d'un patient de 79 ans qui serait hypertendu mal suivi, qui a été reçu pour ischémie du membre supérieur droit allant des doigts au tiers proximal de l'avant-bras droit (Figure 4).

**Chronologie:** ce tableau serait apparu progressivement une semaine après une hospitalisation dans un poste de santé en région pour fièvre associée à une toux, une asthénie intense et des courbatures sans signes respiratoires. Un test *PCR* réalisé était positif. Un traitement médical a été instauré.

**Figure 5:**
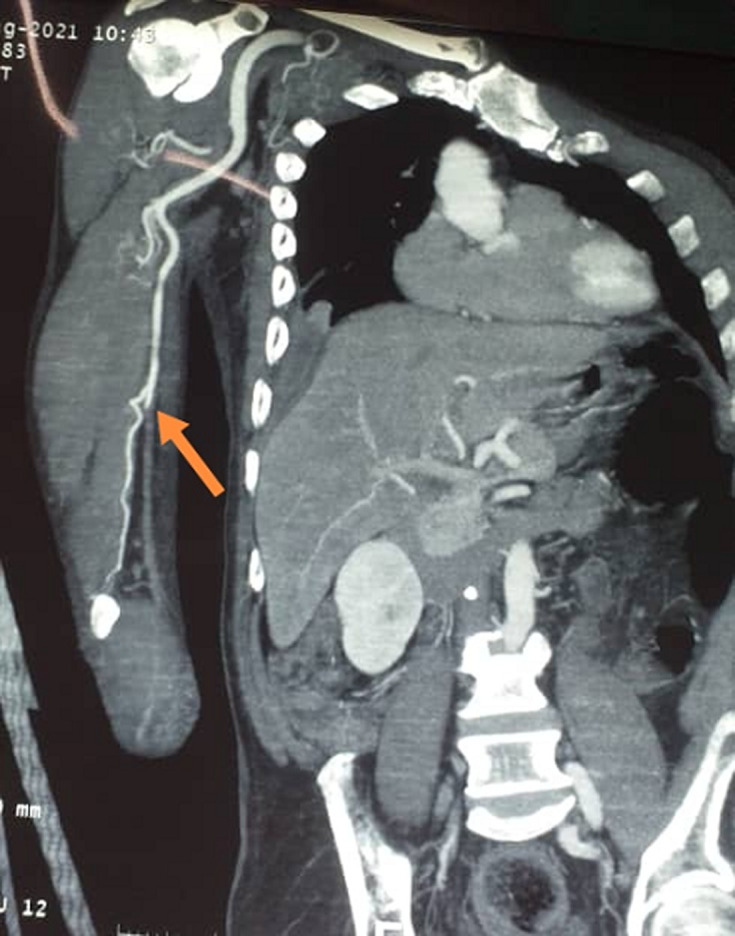
angio TDM montrant une interruption du produit de contraste

**Démarche diagnostique:** le scanner pulmonaire ne montrait pas d'atteinte du parenchyme. L'angioscanner du membre supérieur montrait une occlusion extensive de l'artère humérale (Figure 5). Les autres examens complémentaires à la recherche de pathologies sous-jacentes (diabète, maladie cardiaque, décompensation de l'hypertension artérielle) étaient revenus normales.

**Figure 6:**
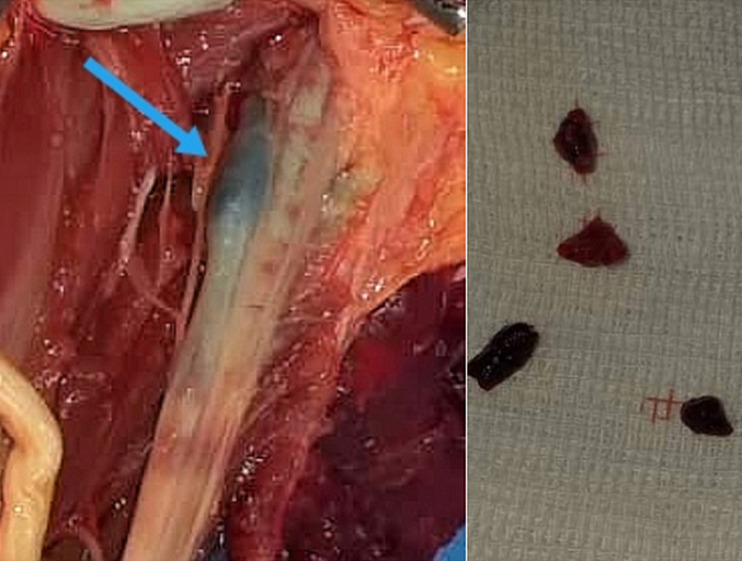
thrombus en peropératoire

**Traitement, suivi et résultats:** une amputation au bras était réalisée et des thrombus ont été retrouvés dans l'artère humérale en peropératoire (Figure 6). Les suites opératoires étaient simples. Il était ensuite admis en unité d'hospitalisation COVID de l'hôpital.

**Consentement du patient:** les auteurs certifient avoir obtenu un consentement oral et signé du patient pour que les images et autres informations cliniques soient rapportées dans le journal. Nous nous abstiendrons à dissimuler son identité mais l'anonymat ne peut être garanti.

## Discussion

Les circonstances de découverte de la COVID-19 peuvent être ses propres manifestations cliniques confirmées par un test *PCR* qui met en évidence l'acide ribonucléique (ARN) virale ou une tomodensitométrie (TDM) pulmonaire en montrant des images en ver dépoli, mais elles peuvent être également ses propres complications. Du fait de la variabilité des tableaux cliniques, de la sensibilité faible des tests *PCR* voire même sa non disponibilité, des cas de errance diagnostique ont été notés et le diagnostic n'a été fait qu'au stade de complications thromboemboliques [3]. Il a été démontré que le nouveau SARS-CoV-2 interagit avec l'enzyme de conversion de l'angiotensine 2 (ACE2). Cette liaison de la protéine virale spike à l'ACE2 diminue l'expression de cette enzyme et active le système rénine-angiotensine (SRA) qui produit l'angiotensine 2, un peptide vasoconstricteur et pro-inflammatoire. L'activation du SRA favorise l'adhésion et l'agrégation plaquettaires et augmente le risque thromboembolique [4].

Dans la littérature l'incidence des événements thromboemboliques (embolie pulmonaire et thrombose veineuse profonde) varie entre 1,6 et 2,4% chez les patients aigus hospitalisés et de 3,3 à 31% chez les patients critiques [5]. Un parmi nos 2 patients était dans un état critique. Beaucoup d'auteurs ont publiés des cas isolés de complications thromboemboliques liés à la COVID-19 [6-8]. Partant de tous ces éléments en plus de l'alitement provoqué par la maladie, il semble évident que nos malades ont présenté une complication liée l'infection à COVID-19 d'autant plus qu'ils ne présentaient aucun comorbidité. L'âge avancé est un facteur de mauvais pronostic. Dans une série de 6 malades qui ont présenté des embolie pulmonaires avec COVID 4 étaient âgés de plus de 50 ans [5]. Dans 2 autres cas isolés de coagulation intravasculaire disséminée dans COVID-19 [6] et de phlegmasie cerulea dolens [8] les patients étaient âgés successivement de 63 et 53 ans. Cette survenue plus fréquente en âge avancé serait liée à leur fragilité vasculaire, leur alitement plus facile par rapport aux sujets jeunes, leur vulnérabilité face à la COVID-19 et leur probabilité plus élevée de présenter des comorbidités. Aucune prédominance par rapport au sexe n'a été rapportée dans la littérature [5].

Dans notre série nous avons un homme et une femme. Les manifestations thromboemboliques sont diverses et variées tous les organes peuvent être touchés : poumon [5], intestin [7], membre inférieur [9], rein, peau [10]. Il est important de signaler que nous n'avons pas retrouvé d'atteintes du membre supérieur dans la littérature. Nos 2 patients ont été reçus au stade d'ischémie dépassée ce qui eut comme conséquence directe une amputation au bras. Une consultation précoce, la disponibilité immédiate des spécialistes et des moyens d'aide au diagnostic (échodoppler, angioTDM, D-Dimer…) et la disponibilité d'un consensus de protocole pour la prise ne charge de COVID-19 aurait pu nous éviter d'en arriver à ce stade et de proposer un traitement adapté et conservateur des membres. Des cas mortalités suites à ses complications n'ont pas été rapportés dans la littérature même s'il faut préciser que nous ne disposons de recul sur les malades des différentes séries pour connaitre leur évolution ultérieure et les séquelles qu'ils ont gardées.

## Conclusion

L'infection au SARS-CoV-2 semble être une pathologie vasculaire par plusieurs mécanismes physiopathologiques, incluant une hyper coagulabilité du sang directe et indirect par l'inflammation induite par la réponse systémique à l'infection. L'expression clinique va de l'atteinte veineuse à l'atteinte artérielle. La COVID-19 apparait être un facteur de risque indépendant des maladies vasculaires, et une meilleure compréhension de la physiopathologie des troubles déboucheront de meilleures stratégies préventives et curatives.
